# Acupuncture for allergic rhinitis: a systematic review and meta-analysis

**DOI:** 10.1186/s40001-022-00682-3

**Published:** 2022-04-25

**Authors:** Min He, Weishan Qin, Zongshi Qin, Changqing Zhao

**Affiliations:** 1 Department of Ophthalmology, Shanxi Medical University Second Affiliated Hospital, No. 382, Wuyi Road, Taiyuan, 030001 Shanxi People’s Republic of China; 2 Department of Acupuncture and Neurology, Guang’anmen Hospital, China Academy of Chinese Medical Sciences, Beijing, People’s Republic of China; 3 Department of Otolaryngology, Shanxi Medical University Second Affiliated Hospital, Taiyuan, Shanxi People’s Republic of China

**Keywords:** Acupuncture, Allergic rhinitis, Meta-analysis, Randomized controlled trials

## Abstract

**Background:**

In this study, we attempted to assess the efficacy and safety of acupuncture for allergic rhinitis (AR), and to test the robustness of the estimated effects.

**Methods:**

The Cochrane methodology standard was followed to conduct this systematic review. Randomized controlled trials (RCTs) comparing acupuncture with other therapies for AR were included. Furthermore, trial sequential analysis was conducted to test the robustness of pooled results. Thirty trials with 4413 participants were included.

**Results:**

Acupuncture improved the nasal symptoms on Total Nasal Symptom Score (TNSS) and quality of life measured by Rhinoconjunctivitis Quality of Life Questionnaire (RQLQ) in adults with AR, compared to acupuncture with no intervention. Acupuncture was also shown to be more effective than sham acupuncture for nasal symptom (RQLQ subscale, *n* = 489, MD − 0.60, 95% CI − 1.16 to − 0.04) and quality of life (RQLQ, *n* = 248, − 8.47 95% CI − 14.91, − 2.03). No clear difference was observed between acupuncture and cetirizine or loratadine. Interestingly, trial sequential analysis (TSA) failed to confirm the aforementioned results. The effect of acupuncture for children/adolescents with AR remains unclear due to insufficient data. The performance bias and attrition bias are serious in most studies that were included. Selection bias may also have affected the quality of the evidence.

**Conclusion:**

Acupuncture may have an advantage over no intervention and sham acupuncture in improving nasal symptoms and quality of life for adults with AR. The effect of acupuncture and cetirizine or loratadine for AR may be similar. Additional trials are necessary to confirm these results.

**Supplementary Information:**

The online version contains supplementary material available at 10.1186/s40001-022-00682-3.

## Introduction

Allergic rhinitis (AR) is a symptomatic nasal disorder resulting from an IgE-mediated immunological reaction to allergen exposure [1]. As a worldwide health problem, AR is now estimated to affect nearly 1.4 billion people globally and continues to be on the rise [2]. Although AR is not a life-threatening illness, it underlies many complications such as bronchial asthma, sinusitis, nasal polyps, otitis media, and allergic conjunctivitis, which affect quality of life and work productivity [3, 4]. The current mainstream management of AR primarily includes allergen avoidance and pharmacotherapy such as topical steroids, oral antihistamines and immunotherapy [5]. These treatments are recommended by the National Guideline Clearinghouse (NGC) as they can rapidly relieve the nasal symptoms. Unfortunately, unpleasant side effects still limit their application. These include epistaxis, dry eyes, and sedation among others. Moreover, some patients prefer non-pharmacologic therapies [5].

Acupuncture was developed from Traditional Chinese Medicine (TCM) techniques. It utilizes acupuncture points, to stimulate lines of meridians that correspond to the flow of energy through the body [6]. Acupuncture is used by approximately 18% of patients with AR [7–9]. Evidences also have demonstrated that acupuncture may modulate biomarkers, including down-regulation of substance P (SP), vasoactive intestinal peptide (VIP), and total IgE to relieve the symptoms of AR [10, 11]. From previous meta-analyses that evaluated the effect of acupuncture on AR, no consistent conclusions have been drawn due to insufficient sample size used in these studies [10, 12, 13]. In 2015, the American clinical practice guidelines on allergic rhinitis listed acupuncture as an optional therapy for AR. However, there is little evidence of RCTs comparing acupuncture with traditional medical therapy. Several systematic reviews relevant on this topic have been published. Unfortunately, they are out-of-date and with concerns of insufficient data [10, 12, 13]. By adding more trials, we aim to update previous evidences from RCTs that have evaluated the efficacy and safety of acupuncture for AR.

## Methods

The systematic review was performed in accordance with the Cochrane Handbook for Systematic Reviews of Interventions and was reported in compliance with the PRISMA statement (see Additional file 1).

### Inclusion criteria

Studies meeting the following criteria were included: (i) randomized controlled trials (RCTs); (ii) participants with a diagnosis of AR (according to validated diagnostic criteria [14]); (iii) intervention includes acupuncture such as manual acupuncture, electrical stimulation (EA) and warm needling (involving the burning of mugwort on an acupuncture needle inserted into the skin to heat the needle); (iv) comparisons are described as follows: acupuncture versus no intervention; acupuncture versus sham acupuncture; acupuncture versus specific western medication; and acupuncture combined with western medication versus western medication alone. Non-English papers were excluded. Primary outcomes were: (i) achieving clinical response in nasal symptoms: defined as the decrease rate of Total Nasal Symptom Score (TNSS) at least 25% [15] or 20% [16] and other definitions stated in the original studies; (ii) any change in nasal symptoms score: TNSS; and (iii) quality of life, measured by any validated scales, such as Rhinoconjunctivitis Quality of Life Questionnaire (RQLQ). Secondary outcomes included: (i) adverse events and (ii) immune responses such as the changes in serum levels of IgE, interferon-γ and interleukin.

### Data sources

Relevant trials were searched on February 18th 2018 using the following databases: Pubmed, The Cochrane Library, EMBASE via Ovid SP, and CBM. The search strategy for each database is presented in Additional file 2.

### Selection of studies

Two reviewers (HM and QZS) independently performed the screening. Titles and abstracts of all searched trials were first screened, then full texts of potentially relevant publications were obtained and inspected. Disagreements between two reviewers were resolved by discussion, with the assistance of a third reviewer (ZCQ) when necessary.

### Data extraction

Data from each study were extracted independently by two reviewers (QZS and HM) using a pre-specified data extraction form. The following information was extracted: first author, publication date, diagnosis and age of participants, treatment duration and management of interventions, study sample size, and characteristics about outcomes such as definition, time points of measurement, and numeric data. Information with a risk of bias was also identified and extracted from eligible studies. Any disagreements were resolved by discussion.

### Risk of bias assessment

We assessed the risk of bias using the methods endorsed by The Cochrane Collaboration [17]. Two reviewers (HM and QZS) independently performed the assessment. Any disagreements were resolved by discussion.

### Statistical analysis

Risk ratios (RR) and mean differences (MD) were used for dichotomous outcome data and continuous outcome data, respectively. *p* < 0.05 was considered statistically significant. Random-effect model was utilized to pool the data. Trial sequential analysis (TSA) was conducted for primary outcomes to test the robustness of the synthetic results. For the dichotomous outcomes, the required information size (RIS) was calculated by using the risk of an event in the control group. The analysis was based on a relative risk reduction of 20% [18], a two-side alpha of 0.05 and beta of 0.20.

An I^2^ estimate ≤ 50% accompanied by a statistically significant Chi^2^ statistic (*p* < 0.1) was interpreted as evidence of substantial levels of heterogeneity [17]. Heterogeneity was investigated following the method in the Cochrane Handbook, Chapter 9.5.3. Post hoc subgroup analysis was performed based on the different countries in which participants were from and the different time points of measurement.

### Assessment of reporting biases

A funnel plot was used to assess publication bias when the included study in one meta-analysis was more than 10 [17].

## Results

### Literature screening

**Fig. 1:**
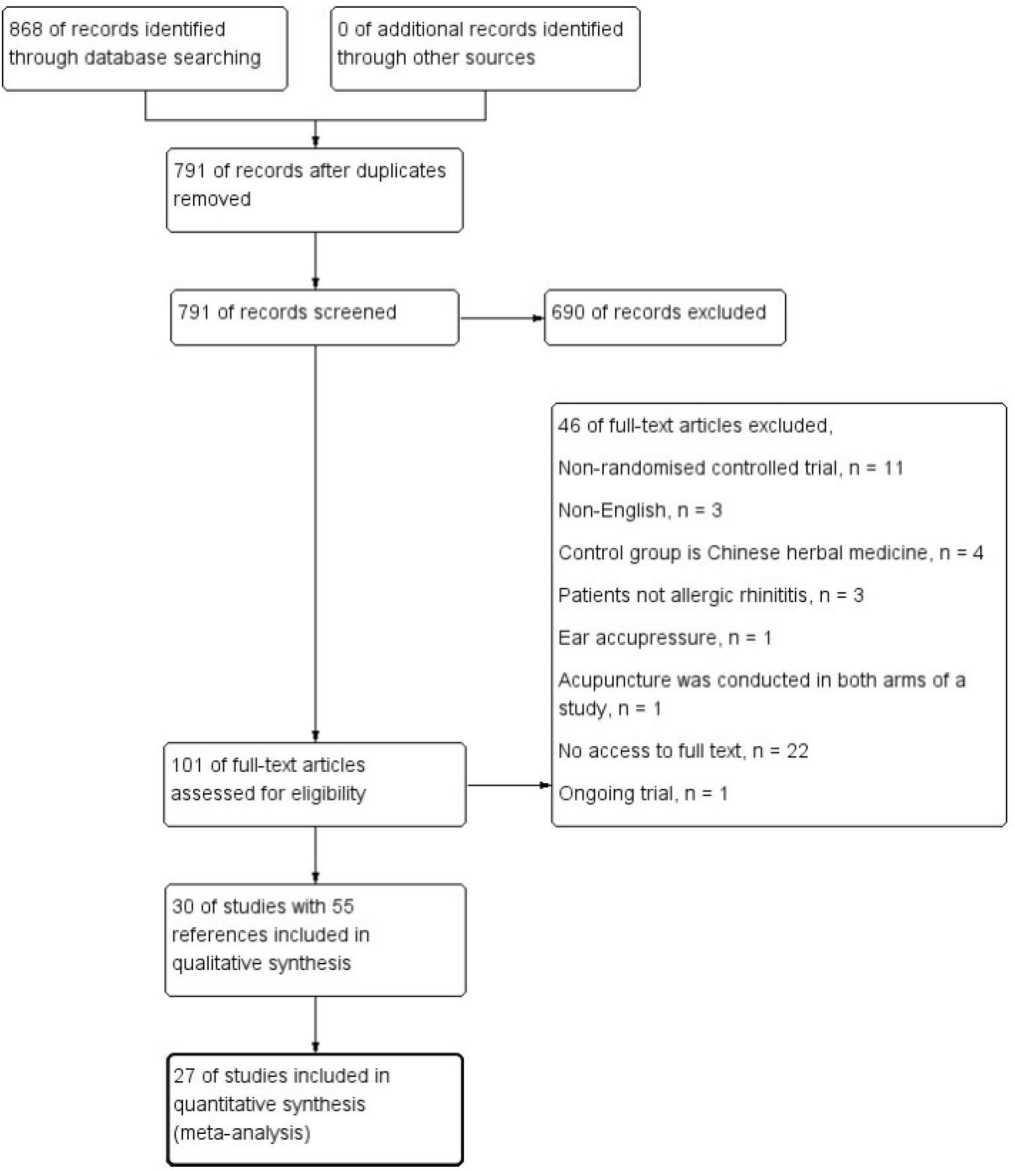
Study flow diagram

Literature search produced 868 references. After removal of duplicates, 791 references were screened. From these, 690 references were excluded according to their titles and abstracts. 101 references were further inspected, among which 46 references were excluded after full-text screening due to no access to full reports, non-randomized design, or ineligible patients and interventions. 55 companion reports [16, 19–76] from thirty trials were finally selected in this review (Fig. 1).

### Characteristics of studies

The 30 trials had 4413 participants in total. The trials were conducted in multiple countries including Australia (3 trials, 356 participants), China (22 trials, 2360 participants), Germany (3 trials, 1427 participants), South Korea (1 trial, 238 participants) and Sweden (1 trial, 32 participants). The sample size of studies ranged from 24 to 981. The treatment durations ranged from 2 to 12 weeks. Acupuncture techniques included needle acupuncture, warm needling, and electroacupuncture; and the controls included no treatments (waiting to receive other interventions at the end of trial), sham acupuncture, cetirizine, loratadine, terfenadine, and desloratadine (Table 1).

**Table 1:** Characteristics of studies

Source	Country	Participants	Trial duration	Intervention	Size	Control	Size	Trial size	Outcomes
Ortiz 2014	Germany	SAR; 16–45 years old	Treatment duration: 8 weeks; follow-up: 52 weeks	Manual acupuncture + cetirizine; 12 sessions over 8 weeks	212	Control 1: sham acupuncture + cetirizine: sham acupuncture treatment entailed superficially inserting fine needles (≤ 20 mm in length) at predefined, distant non-acupuncture points bilaterally; acupuncture points were the same with real acupuncture group; 12 sessions over 8 weeks; control 2: cetirizine dihydrochloride	102/108	422	RQLQ; RMS; QoL (SF-36);
Brinkhaus 2008	Germany	Seasonal and/or perennial AR; ≥ 18 years old	Treatment duration:3 months; follow-up: 6 months	Acupuncture: the number of needles and the acupuncture points used were chosen at the physicians’ discretion; 15 times 3 months	487	No acupuncture on the wait-list	494	981	RQLQ; QoL (SF-36); clinical improvement; adverse events
Chen 2015	China	Moderate-to-severe AR; 22–60 years old	Treatment duration: 8 weeks; follow-up: 3 months	Manual acupuncture; three times a week	34	Cetirizine hydrochloride; 10 mg per day	32	66	Clinical improvement; TNSS; adverse events
Choi 2012	Korea and China	Moderate to severe PAR; > 18 years old	Treatment duration: 4 weeks; follow-up: 9 weeks	Manual acupuncture; three times per week	97	Control 1: Sham acupuncture: needles were inserted at non-acupuncture points that were 1–1.5 cm away from the acupuncture sites. The needles were inserted to a depth of 3–5 mm using a shallow needling technique; three times per week; control 2: waiting list	94/47	238	TNSS; TNNSS; RQLQ
Ng 2004	Hong Kong	Persistent AR; children; 11.7 ± 3.2 years old	Treatment duration: 8 weeks; follow-up: not reported	Manual acupuncture; twice per week	35	Sham acupuncture: the needle was inserted to a depth of only 0.3 cm; twice per week	37	72	Clinical improvement; serum immunological indicators; medication score; daily relief; adverse events
Hauswald 2014	German	PER; age: mean 28.1 years old, sd 9.9 years	Treatment duration: 6 weeks; follow-up: 16 weeks	Acupuncture; twice per week	15	Loratadine: 10 mg/d; treatment duration: 21 days	9	24	Allergic parameters; interleukin profiles
Huang 2012	China	AR; 16–65 years old	Treatment duration: 30 days; follow-up: not reported	Acupuncture; once a day	40	Loratadine; 10 mg/d	36	76	Clinical improvement; adverse events
Li 2003	China	AR; 7–65 years old	Treatment duration: 30 days; follow-up: not reported	Acupuncture; once a day	63	Cetirizine; 10 mg/time, three times a day	35	98	Clinical improvement
Li 2007	China	AR; 5–68 years old	Treatment duration:1 month; follow-up: not reported	Acupuncture + electroacupuncture; once per day	50	Cetirizine: 10 mg/d; three times a day	50	100	Clinical improvement; serum immunological indicators
Li 2013	China	Moderate to severe AR; 18–70 years old	Treatment duration: 30 days; follow-up: 6 months	Acupuncture + warm needling; once per day	62	Terfenadine tablets; 60 mg/time; 2 times per day, oral taken	62	124	Clinical improvement
Li 2015	China	AR; 18–63 years old	Treatment duration: 20 days; follow-up: not reported	Loratadine + acupuncture	50	Loratadine: 10 mg/d	50	100	Clinical improvement
Magnusson 2004	Sweden	SAR; age: 18–50 years old	Treatment duration: 12 treatments; follow-up: not stated	Manual acupuncture	18	Sham acupuncture: needles were placed subcutaneously, 1 or 2 cm away from the true acupuncture point, no deqi was elicited	14	32	IgE
McDonald 2016	Australia	Moderate to severe AR; 18–45 years old	Treatment duration:8 weeks; follow-up: 4 weeks	Manual acupuncture; twice weekly	49	Control 1: sham acupuncture, to a depth of 5 to 10 mm and 10 to 15 mm, respectively; twice weekly; control 2: no acupuncture	52/50	151	Serum immunological indicators; iTNSS; mini-RQLQ; adverse events
Ni 2006	China	AR; 9–58 years old	Treatment duration: 1 week; follow-up: not reported	Acupuncture; once a day	195	Tranilast capsules; 0.2 g/time, three times a week	191	386	Clinical improvement
Ou 2014	China	AR; 9–55 years old	Treatment duration: 20 days; follow-up: not reported	Acupuncture; once a day	33	Desloratadine dispersible tablets; 5 mg/d	33	66	Clinical improvement
Rao 2006	China	AR; 20–66 years old	Treatment duration: 28 days; follow-up: 6 months	Acupuncture; once a day	50	Control 1: ear acupoint; 3 to 5 times per day; control 2: cetirizine 10 mg/d	50/50	150	Clinical improvement; serum immunological indicators
Shi 2013	China	Moderate to severe persistent AR; 18–60 years old	Treatment duration: 4 weeks; follow-up: not reported	Acupuncture electroacupuncture; three times a week	20	Cetirizine hydrochloride; 10 mg/d	16	36	TNSS
Wang 2013	China	Moderate to severe persistent AR; 19–58 years old	Treatment duration: 4 weeks; follow-up: not reported	Acupuncture + electroacupuncture; three times a week	30	Cetirizine: 10 mg/d	30	60	TNSS; adverse events
Wang 2013a	China	AR; 18–60 years old	Treatment duration:4 weeks; follow-up: 3 month	Acupuncture electroacupuncture; three times a week	41	Loratadine: 10 mg/d	40	81	Clinical improvement; adverse events
Wang 2014	China	moderate to severe AR plus persistent allergic rhinitis; 18–60 years old	Treatment duration: 4 weeks; follow-up: 4 weeks	Acupuncture; three times a week	120	Cetirizine hydrochloride; 10 mg/d	120	240	–
Wang 2015	China	AR; 13–45 years old	Treatment duration: 1 month; follow-up: not reported	Acupuncture; once per day	30	Loratadine: 10 mg/d	30	60	Clinical improvement
Wang 2016	China	AR; 23–70 years old	Treatment duration: 2 weeks; follow-up: not reported	Acupuncture + moxibustion; once per day	30	Loratadine: 10 mg/d	30	60	Clinical improvement
Xie 2013	China	AR; 20–65 years old	Treatment duration: 1 month; follow-up: not reported	Acupuncture; once per day	30	Loratadine: 10 mg/d	30	60	Clinical improvement
Xie 2015	China	AR; 18–60 years old	Treatment duration:60 days; follow-up: not reported	Sanfu tian warming needle moxibustion; 30 times per year	80	Control 1: not Sanfu tian warming needle moxibustion; 30 times per year; control 2: cetirizine 10 mg/d	80/80	240	Clinical improvement; serum immunological indicators; RQLQ
Xue 2002	Australia	Seasonal AR; 18–70 years old	Treatment duration: 4 weeks; follow-up: 4 weeks	Manual acupuncture: 10–40 mm in depth; three times per week	17	Sham acupuncture: 0.26 mm in diameter and 15 mm in length, 1.5 cm lateral to the related points; three times per week	13	30	FPS symptom; RMS; adverse events
Xue 2007	China	AR; 16–70 years old	Treatment duration:8 weeks; follow-up: 12 weeks	Real (manual) acupuncture depth of 10–30 mm transversely, obliquely or perpendicularly; twice per week	42	Sham acupuncture: the insertion sites were 1–1.5 cm from the acupoints; twice weekly	38	80	TNNS; adverse events
Xue 2015	Australia	SAR; 16–70 years old	Treatment duration: 4 weeks; follow-up: 8 weeks	Manual acupuncture; three times per week	88	Sham acupuncture: the points that were needled were located on non-acupoints area beside the real acupoints; three times per week	87	175	Symptom severity; RQLQ; global change
Miao 2015	China	AR; 7–65 years old	Treatment duration: 4 weeks; follow-up: 12 months	Acupuncture; six times a week	14	Loratadine:10 mg/d	12	26	Clinical improvement; recurrence rate
Zhao 2012	China	AR; 15–74 years old	Treatment duration:4 weeks; follow-up: not reported	Ebastine + acupuncture; once per week	52	Ebastine: 10 mg/d	49	101	Clinical improvement; serum immunological indicators
Zhu 2014	China	AR; 18–46 years old	Treatment duration: 2 weeks; follow-up: not reported	Acupuncture + electroacupuncture + antihistamine or glucocorticoid; once per day	39	Antihistamine or glucocorticoid	39	78	Clinical improvement; adverse events

*AR* allergic rhinitis, *RQLQ* Rhinoconjunctivitis Quality of Life Questionnaire, *RMS* rescue medication score, *FPS* five-point scale, *TNSS* Total Nasal Symptom Score, *SF-36* short-form 36, *TNNSS* total non-nasal symptoms score

### Risk of bias

**Fig. 2:**
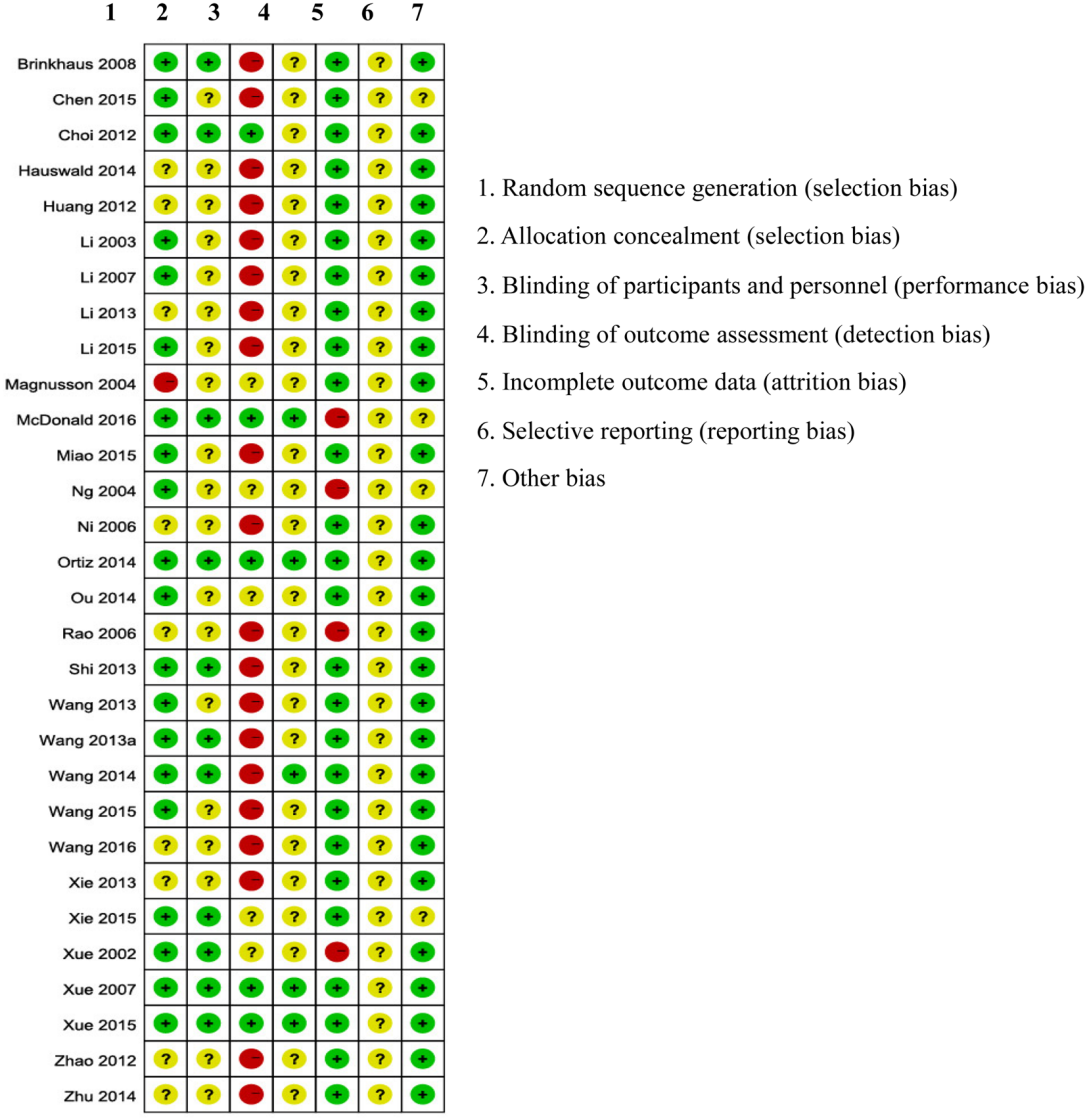
Risk of bias graph

Of the 30 trials included in this study, 20 provided sufficient information on randomization and were rated as low risk of selection bias. The methods of randomization included central randomization (5 trials), random number table (8 trials), and computer generated random number sequence (7 trials). One trial enrolled only 32 participants and used a coin toss to assign participants. This trial was rated as high risk of bias [48]. Nearly two-thirds of the trials did not report procedures to conceal the allocation scheme and 67% of the included trials were rated as high risk of bias in blindness of participants and personnel enrolled. Only 17% of the included trials stated that the outcome assessors were blinded. Other studies did not report such information. Four studies were rated as high risk of bias because of incomplete data. The judgment was based on the facts that the participants dropped out from the trials due to either low efficiency, adverse events, or high attrition rate and imbalance between groups. Selective report was rated as an unclear risk of bias for all included studies, as it was not possible to obtain the protocols of these studies (Fig. 2).

### Allergic rhinitis in adults

#### Acupuncture versus no intervention (on the wait-list)

**Fig. 3:**
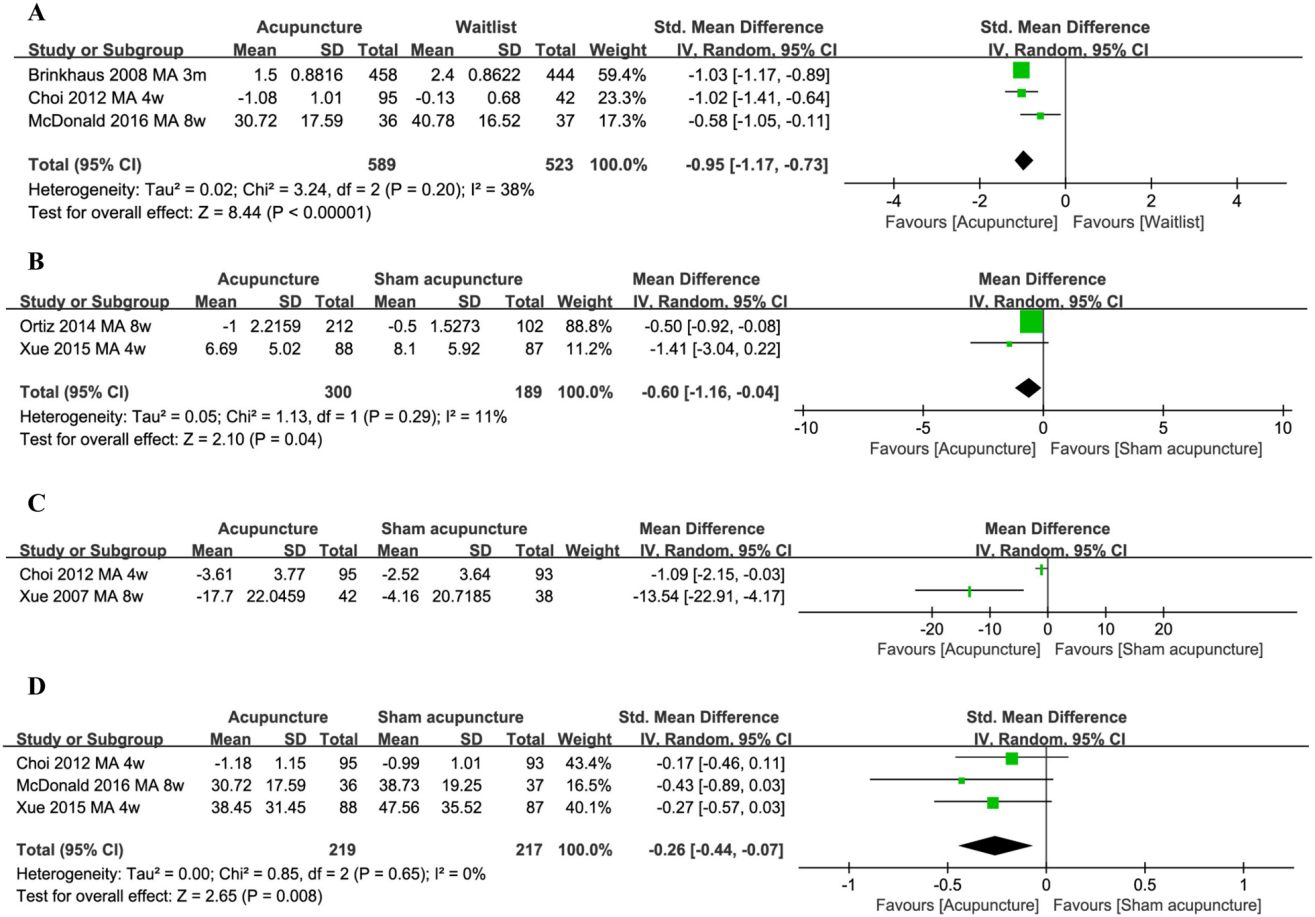
Acupuncture compared to distinct controls. **A** Acupuncture vs. no treatment QOL; **B** acupuncture vs. sham acupuncture nasal symptoms (RQLQ); **C** acupuncture vs. sham acupuncture nasal symptoms (TNSS); **D** acupuncture vs. sham acupuncture QOL

Three trials from seven companion reports compared acupuncture with no intervention control [11, 26, 31, 42, 43, 47, 77]. Choi 2012 [31, 42, 43, 47, 77] found that compared to no intervention control, acupuncture relieved the severity of total nasal symptoms score on TNSS scale (Additional file 3: 1.1). Data pooled from three studies also showed that acupuncture improved the life quality of patients, measured by Rhinoconjunctivitis Quality of Life Questionnaire (RQLQ) or Mini RQLQ (*n* = 1112, SMD − 0.95, 95% CI − 1.17, − 0.73, Fig. 3A). Subgroup analyses showed this beneficial effect of acupuncture was observed in all time points of outcome measurement (4 weeks, 8 weeks and 3 months) and in different countries (Germany, China, Korea and Australia) (Additional file 4: 1.1 and 1.2). Two studies reported adverse events relevant to acupuncture. One study reported two patients complaining of papules, pruritus, ocular pruritus and subcutaneous bleeding in the acupuncture group, while no adverse events occurred in the wait-list group [31]. The other study reported approximately a 3% incidence rate of adverse events in the acupuncture group. These included slight bruising, acute transitory pain upon needle insertion, acute transitory pain and pins, soreness, itching, swelling and tingling [11]. With regard to immune responses, data from a single study concluded that there was no difference in serum IgE level between acupuncture and no-intervention control (Additional file 3: 1.2). This study also concluded that the levels of testing cytokines, neuropeptides and neurotrophins had no difference between acupuncture and no intervention control after 12 weeks of intervention. Due to insufficient data, the subgroup analysis for adverse events was not applicable.

#### Acupuncture versus sham acupuncture

Four trials in 9 companion reports compared acupuncture with sham acupuncture [11, 26, 31, 42, 43, 48, 69, 70, 77]. Sham acupuncture refers to a shallow needling technique in which needles were inserted 10 to 15 mm away from the actual acupuncture points. The post-intervention nasal symptoms score was lower in the acupuncture group than in the sham acupuncture group (RQLQ nasal symptom subscale: *n* = 489, MD − 0.60, 95% CI − 1.16 to − 0.04, Fig. 3B). Subgroup analysis showed that this beneficial effect of acupuncture was observed after 8 weeks of intervention and in participants recruited from Germany. However, this effect was not observed after 4 weeks of intervention and in participants recruited from Australia (Additional file 4: 2.1 and 2.2). Choi [31, 42, 43, 48, 77] and Xue [69, 70] used a daily TNSS score and a weekly mean TNSS score to test the nasal symptoms post-intervention. Both studies showed that nasal symptoms were significantly improved in the acupuncture group than the sham acupuncture group (daily TNSS: *n* = 188, MD − 1.09, 95% CI − 2.15 to − 0.03; weekly mean TNSS: *n* = 80, MD − 13.54, 95% CI − 22.9 to − 4.17, Fig. 3C). Due to insufficient data, subgroup analysis for this outcome was not applicable.

Evidence from three trials demonstrated that the acupuncture group had significantly improved life quality (RQLQ) compared to the sham acupuncture group (*n* = 436, SMD − 0.26 95% CI − 0.44, − 0.07, Fig. 3D). Subgroup analysis showed that this beneficial effect of acupuncture was observed after 4 weeks of intervention and in participants recruited from Australia. This effect, however, was not observed after 8 weeks of intervention and in participants recruited from Korea and China (Additional file 4: 2.3 and 2.4). The adverse events of acupuncture included mild discomfort, mild headache, dizziness, pain in needling area, feeling tired after needling among others. Due to the low incidence of these adverse events, no clear difference was found between the acupuncture and the sham acupuncture group for this outcome (Additional file 3: 2.1). The subgroup analysis for this outcome was not applicable due to insufficient data.

#### Acupuncture versus western medication

Seventeen trials [16, 19, 29, 32, 33, 35, 36, 38, 40, 55, 56, 61, 62, 64, 72, 73, 75] compared acupuncture with western medication. The western medication used in trials included cetirizine, loratadine, terfenadine, Tranilast capsules and desloratadine.

**Fig. 4:**
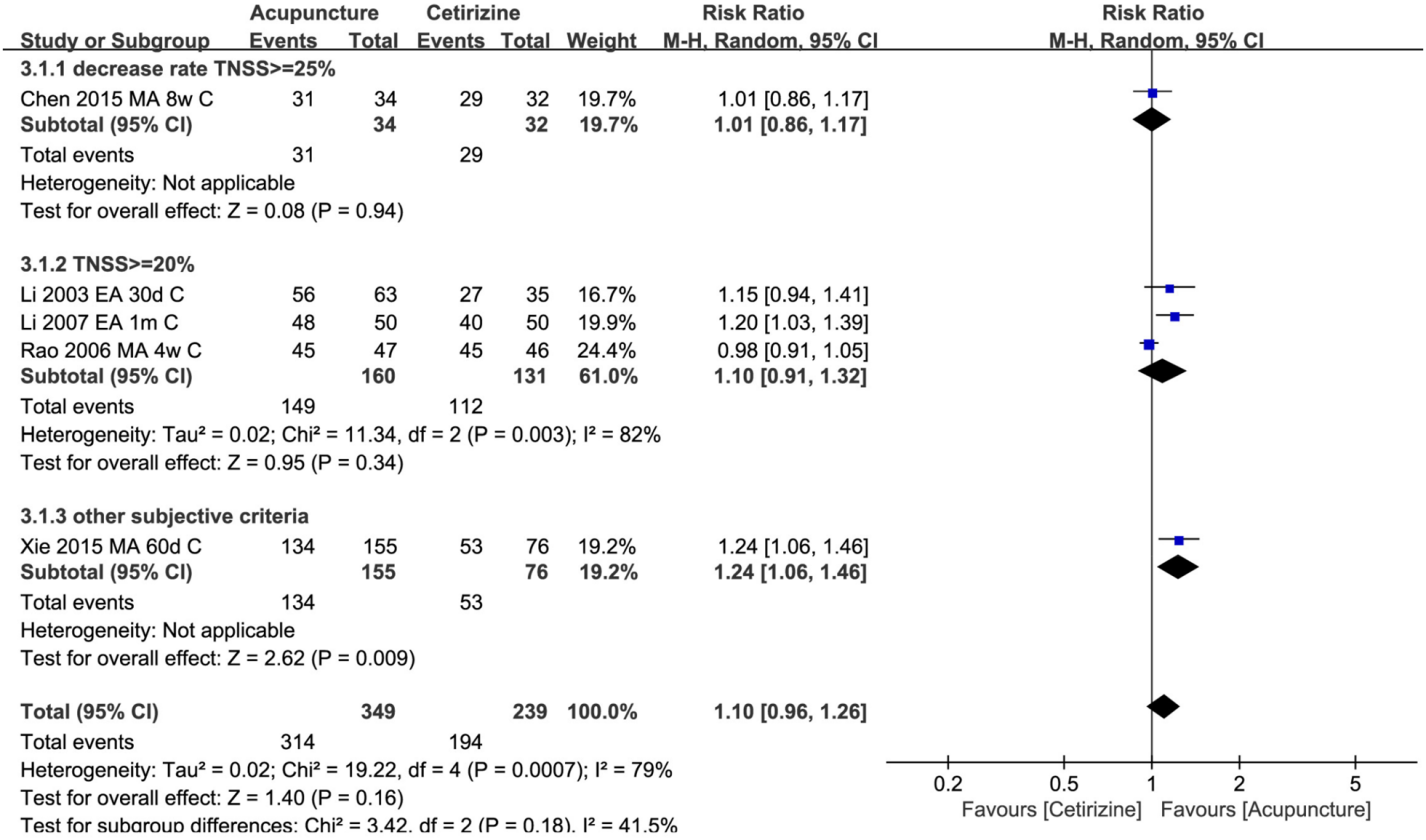
Acupuncture vs. cetirizine: clinical response

**Fig. 5:**
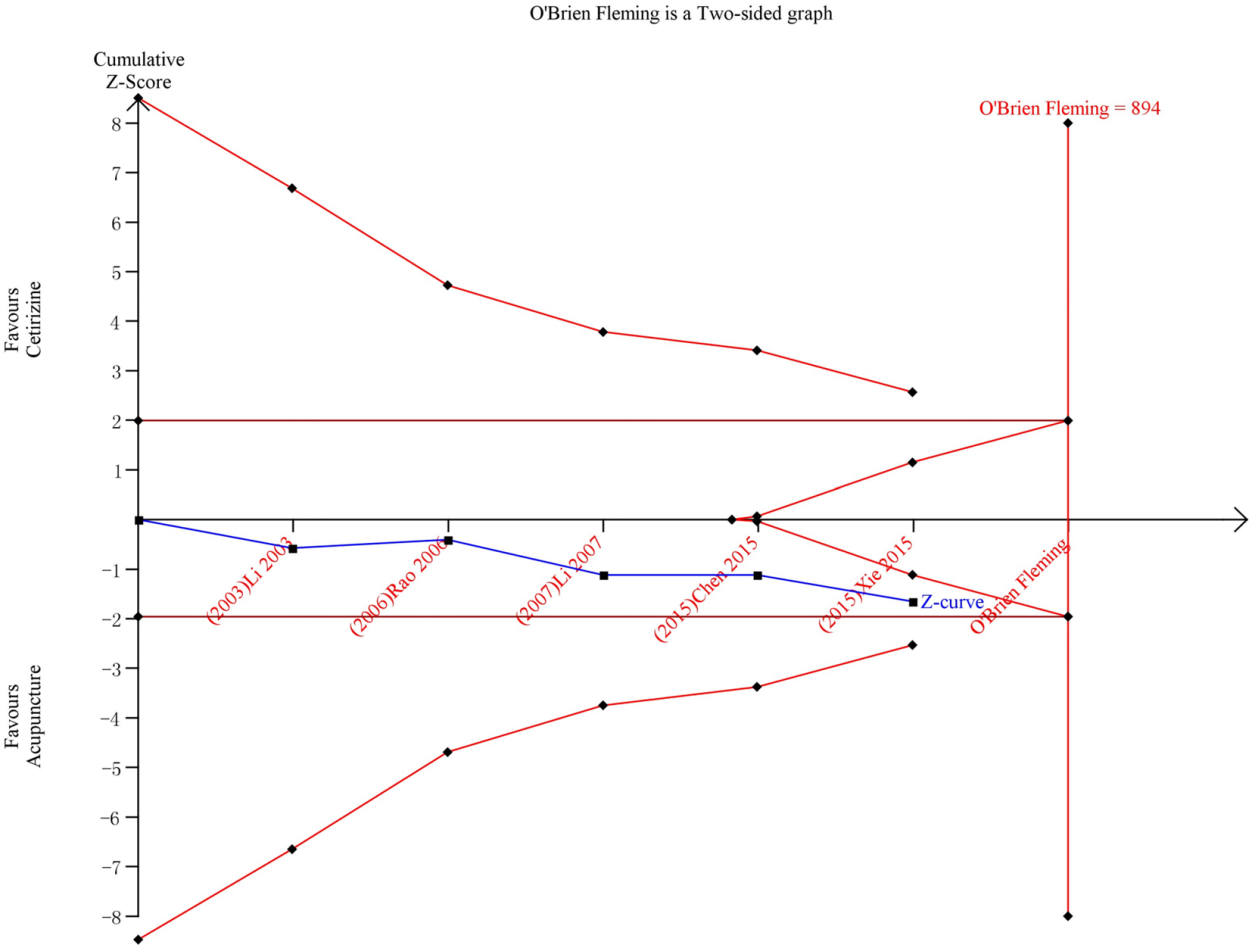
TSA for clinical response—acupuncture vs. cetirizine

Six trials compared acupuncture with cetirizine. All trials were conducted in China. The studies used different definitions of clinical responses. There was no difference for clinical response between these two groups (*n* = 588, RR 1.10 95% CI 0.96, 1.26, Fig. 4). Subgroup analysis showed that no difference for the above outcomes between two groups was observed after both 4 weeks and 8 weeks of intervention (Additional file 4: 3.1). As revealed by the TSA result, this finding was not robust and, therefore, further trials are needed (Fig. 5). No difference was found between the two groups for nasal symptoms (*n* = 214, MD − 0.77, 95% CI − 1.67 to 0.12, Additional file 3: 3.1). Subgroup analysis showed that nasal symptoms were improved in acupuncture group after 8 weeks, but not after 4 weeks (Additional file 4: 3.2). The difference in the quality of life between two groups was inconsistent. Two studies [29, 65] found the acupuncture group having a better quality of life while another study found the opposite result [61]. Due to insufficient data, the subgroup analysis for this outcome was not applicable. Acupuncture reduced IgE levels in serum more than cetirizine. However, this difference was not found for other immune molecules including IL-4 and INF gamma (Additional file 3: 3.2). Due to insufficient data, the subgroup analysis for this outcome was not applicable. One study also measured serum neuropeptides such as, vasoactive intestinal peptide (VIP) and substance P in the acupuncture and cetirizine groups after 1 month of intervention. Results showed that there was no difference between the two groups for neuropeptides [73]. Due to insufficient data, the subgroup analysis for this outcome was not applicable.

**Fig. 6:**
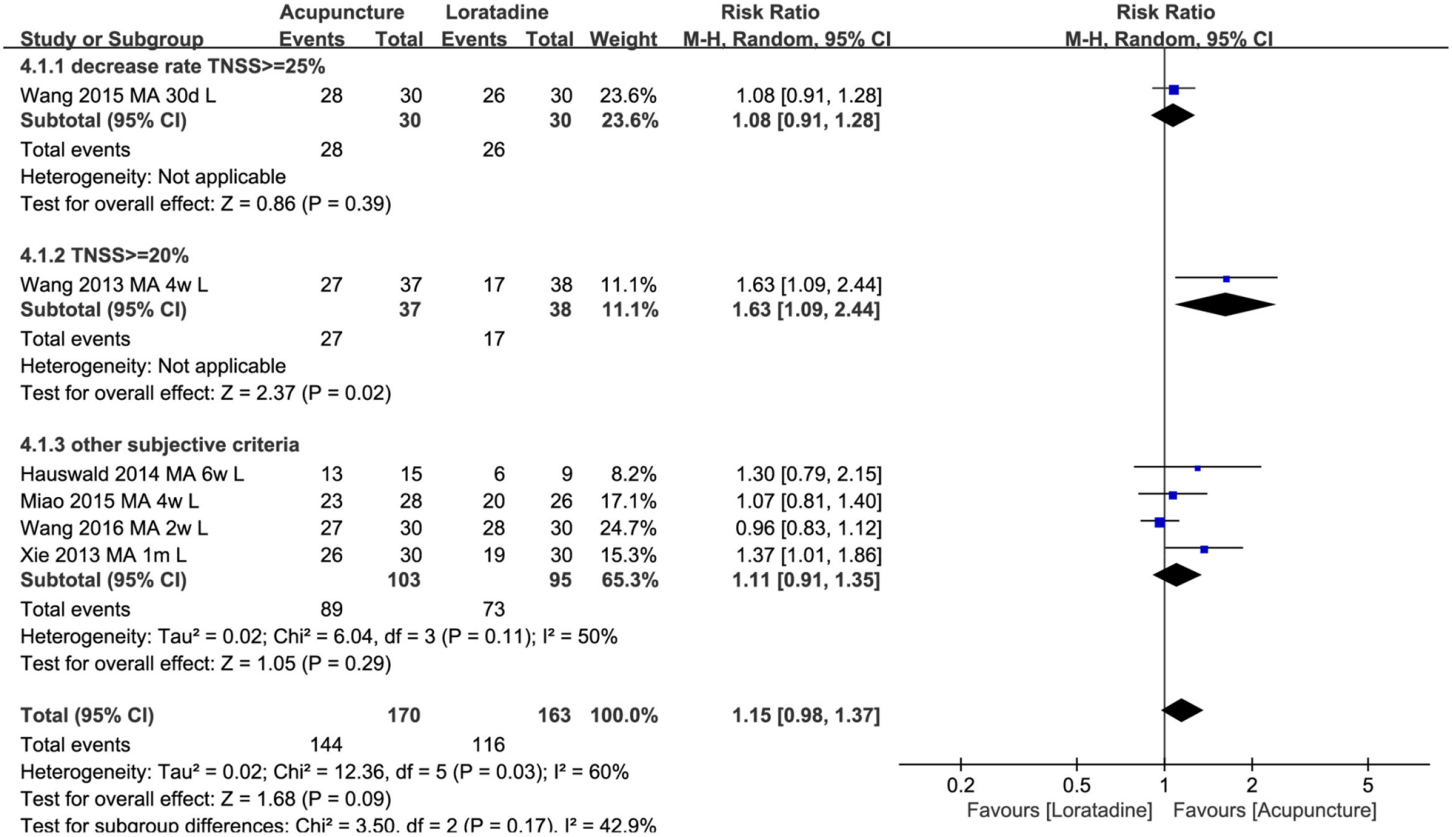
Acupuncture vs. loratadine: clinical response

**Fig. 7:**
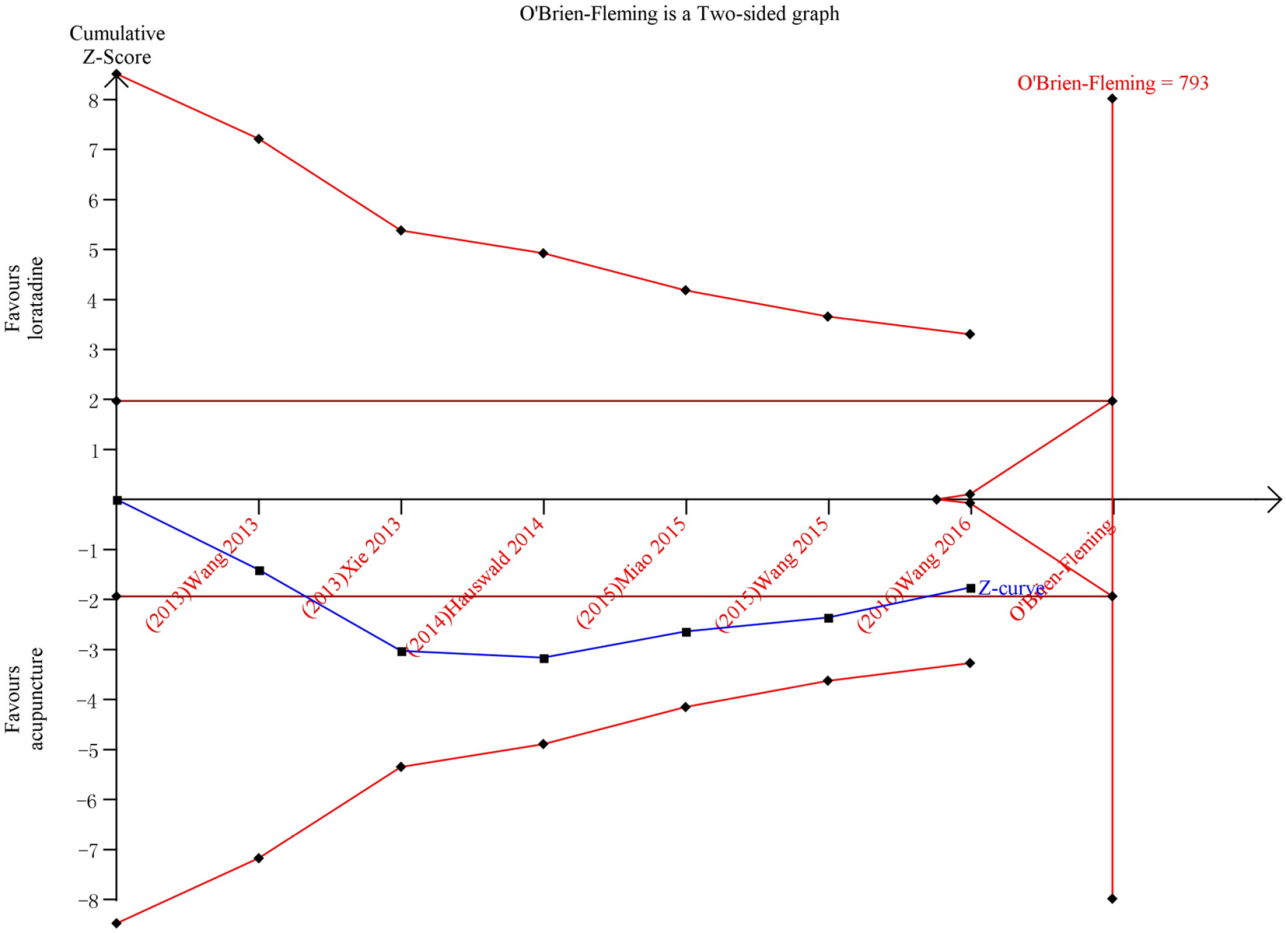
TSA for clinical response—acupuncture vs. loratadine

Six trials compared acupuncture with loratadine. No difference was found between the two groups for clinical response (*n* = 333, RR1.15 95% CI 0.98, 1.37, Fig. 6). As revealed by the TSA result, this finding was not robust and, therefore, more trials are needed (Fig. 7). Subgroup analysis showed that there was no difference for the above outcomes between groups after 2 weeks, 4 weeks and 6 weeks of intervention and in participants recruited from Germany and China (Additional file 4: 4.1 and 4.2). Compared to loratadine, acupuncture improved nasal symptoms and reduced the risk of nasal symptoms and relapse at 1 year (Additional file 3: 4.1 and 4.2).

A single study compared acupuncture with terfenadine, tranilast capsules, and desloratadine dispersible. Results showed that acupuncture led to a higher rate of clinical response than tranilast capsules and desloratadine dispersible (Additional file 3: 6.1 and 7.1). Nasal symptoms were also reduced in the acupuncture group than those in the desloratadine dispersible group (Additional file 3: 7.2). No clear difference was found between acupuncture and terfenadine for the clinical response (Additional file 3: 5.1). Due to insufficient data, the subgroup analysis for this outcome was not applicable.

#### Acupuncture in addition to western medicine vs. western medication alone

Only four trials in 10 companion reports [21, 22, 24, 25, 27, 45, 52, 53, 58, 71] were included in this comparison. Three trials reported a number of participants with a clinical response by using two different definitions (TNSS ≥ 25%; TNSS ≥ 20%). All trials supported that a combination of acupuncture and western medication can lead to a higher proportion of clinical responses than western medication alone [45, 58, 71] (Additional file 3: 8.1). By enrolling 320 participants, Ortiz et al. [21, 22, 24, 25, 27, 52, 53] found that after treatment, nasal symptoms were less severe in the combination group compared to western medication alone (Additional file 3: 8.2). Evidence from one trial 32 showed that the risk of adverse events such as joint pain had no significant difference between the acupuncture plus western medication and the western medication alone group (Additional file 3: 8.3). Due to insufficient data, the subgroup analysis for this outcome was not applicable.

One study found that the levels of certain cytokines, such as IL-4, IL-6 and IL-10 decreased when acupuncture was implemented along with western medication [71]. Another study also found adding acupuncture to medication was advantageous with regard to decreasing the levels of vascular cell adhesion molecule-1, IL-4 and IL-10 [58]. Due to insufficient data, the subgroup analysis for this outcome was not applicable.

### Allergic rhinitis in children

Only two trials enrolled participants younger than 18 years old [20, 49–51]. Ng et al. found no difference between real acupuncture and sham acupuncture in the severity of nasal symptoms (*n* = 72, MD − 1.76, 95% CI − 3.59 to 0.07) (Additional file 3). Additionally, the authors did not detect a difference between groups in incidence of adverse events (Additional file 3). Moustafa et al. compared laser phototherapy with laser acupuncture and found nasal symptoms such as rhinorrhea, nasal obstruction and nasal discharge improved equally in both groups [49]. Due to insufficient data, the subgroup analysis for this outcome was not applicable.

### Publication bias

Due to the small number of included studies, funnel plot was not conducted to test the publication bias. Therefore, publication bias is unclear.

## Discussion

Results showed that for adults with moderate-to-severe AR, acupuncture is better than no intervention in reducing the severity of nasal symptoms and improving the patients’ quality of life. Acupuncture is also superior to sham acupuncture in lowering the severity of nasal symptoms and improving quality of life. As meta-analysis indicates, both acupuncture and western medication improve clinical response of AR. For instance, the clinical response rates (TNSS ≥ 25%) in acupuncture and cetirizine are 91.2% and 90.6%, respectively. However, compared to cetirizine or loratadine, acupuncture did not show an advantage in improving clinical response and relieving nasal symptoms. As revealed by the TSA result, this finding was not robust and, therefore, more trials are necessary to provide more data. Whether acupuncture is better than cetirizine in improving patients’ quality of life remains controversial. Moreover, acupuncture, in addition to western medication may achieve better outcomes (such as higher clinical response rate and better quality of life) than Western medication alone. Acupuncture seems to lower the IgE levels in serum when compared to cetirizine. Whether acupuncture can lower the serum level of other immune molecules, however, remains unclear. As there is lack of big data showing the difference between acupuncture and western medication in the treatment of AR, we conducted this systematic review on current evidences to address this issue. However, the data available currently are underpowered to test a difference between acupuncture and western medication. Acupuncture is more acceptable in the Chinese patients with AR, likely due to their values and preferences to traditional Chinese medicine. Two trials assessed the effect of acupuncture on children with AR. Results from an individual trial showed no difference between acupuncture and sham acupuncture for AR. The data, however, is very limited.

### Comparison with other reviews

Most of the studies that were included have a high risk of performance bias. Selection bias, detection bias and attrition bias are also of some concern, though not serious. We do not attempt to draw any conclusion based on results of subgroup analysis, as the data are insufficient to show the tendency of differences between subgroups. The influence of different treatment durations of acupuncture for AR remains unclear. Whether the effect of acupuncture varies in different countries also requires further exploration. However, the findings of subgroup analysis may provide possible hypothesis for future studies.

Several systematic reviews were published on this topic [10, 12, 13]. In 2015, Feng found 13 RCTs and suggested acupuncture as a safe and valid treatment option for AR patients. However, they combined all other interventions (such as sham acupuncture, no intervention) as one control group. Therefore, the clinical heterogeneity was substantial as different control groups led to different estimates of effects [10]. Another review included seven RCTs suggesting that it was not possible to recommend acupuncture as a proven treatment for AR because of insufficient data [12]. Due to small sample size, one systemic review conducted in 2009 failed to show specific effects of acupuncture for seasonal AR, and the results for perennial AR provided suggestive evidence of the effectiveness of acupuncture [13].

## Limitations

By combining the data with different controls separately, our review reduced the clinical heterogeneity in the control groups. Meanwhile, we used TSA to test the robustness of evidence, which demonstrates the power of our findings. Similar to other studies, this review does have some limitations. Firstly, the clinical heterogeneity in the intervention group is significant. The techniques of acupuncture (such as manual acupuncture, electroacupuncture, or warm needling) and acupuncture points used in individual trials vary as well as the treatment frequency and duration (from 7 days to 12 weeks). All these factors may influence the effect measurement while introducing statistical heterogeneity. Secondly, only few studies assessed the effect of acupuncture on children with AR, which greatly limited the applicability of the evidence. Thirdly, all trials contributing data for acupuncture versus western medication were conducted in China and were with serious risk of bias. These findings should be interpreted very cautiously.

## Conclusion

For adults with AR, acupuncture is superior to no intervention and sham acupuncture in lowering the severity of nasal symptoms and improving the life quality of patients. The effect of acupuncture and cetirizine/loratadine may be similar. Whether acupuncture can decrease the serum level of immune response molecules is still uncertain. The effect of acupuncture on children with AR remains unclear.

Future trials with well-randomized assignments are required. More trials are indeed required to evaluate the effect of acupuncture on children with AR. Further trials are also needed to evaluate the difference between real acupuncture and sham acupuncture for AR, as the data are insufficient at present. When measuring the clinical response, investigators of future trials should utilize objective definitions with a unified minimal clinical importance threshold value. Future studies should aim to explore whether different treatment durations of acupuncture influence the treatment effect and whether the effect of acupuncture varies in patients from different countries.

## Supplementary Information

**Additional file 1.** PRISMA 2009 checklist.

**Additional file 2.** Search strategies and items used in each database.

**Additional file 3.** Meta-analysis result for this review.

**Additional file 4.** Result of subgroup analysis according to different time-points and countries.

## Data Availability

The data are available under request.

## Abbreviations

AR: Allergic rhinitis
CI: Confidence interval
EA: Electrical stimulation
VIP: Vasoactive intestinal peptide
RCT(s): Randomized controlled trial (s)
RR: Risk ratio
RIS: Required information size
RQLQ: Rhinoconjunctivitis Quality of Life Questionnaire
SMD: Standard mean difference
SP: Substance P
TSA: Trial sequential analysis
TNSS: Total Nasal Symptom Score
MD: Mean difference
NGC: National Guideline Clearinghouse
