# Supplementary material for: Acupuncture for allergic rhinitis: a systematic review and meta-analysis
Source: Eur J Med Res. 2022 Apr 25;27:58. doi: 10.1186/s40001-022-00682-3 (PMC9036742; doi:10.1186/s40001-022-00682-3)
Supplement: Supplementary file 2 — Additional file 2. Search strategies and items used in each database. [file 40001_2022_682_MOESM2_ESM.docx]

PubMed

#1 "Rhinitis, Allergic"[Mesh] OR "Rhinitis, Allergic, Perennial"[Mesh] OR "Rhinitis, Allergic, Seasonal"[Mesh]

#2 allergic rhinitides[Title/Abstract] OR allergic rhinitis[Title/Abstract] OR allergic rhinopathy[Title/Abstract] OR eosinophil rhinitis[Title/Abstract] OR eosinophile rhinitis[Title/Abstract] OR eosinophilic rhinitis[Title/Abstract] OR eosinophilous rhinitis[Title/Abstract] OR allergica rhinitis[Title/Abstract] OR eosinophila rhinitis[Title/Abstract] OR pollen allergy[Title/Abstract] OR pollen allergies[Title/Abstract] OR pollinosis[Title/Abstract] OR pollinoses[Title/Abstract] OR hay fever[Title/Abstract] OR hayfever[Title/Abstract] OR pollen hypersensitivity[Title/Abstract] OR pollen sensitivity[Title/Abstract] OR pollenosis[Title/Abstract] OR pollinose[Title/Abstract] OR seasonal rhinitis[Title/Abstract]

#3 #1 OR #2

#4 "Acupuncture"[Mesh] OR "Acupuncture Therapy"[Mesh] OR "Electric Stimulation"[Mesh] OR "Electric Stimulation Therapy"[Mesh] OR "Chronaxy"[Mesh] OR "Electroshock"[Mesh]

#5 acupuncture[Title/Abstract] OR electrical stimulation[Title/Abstract] OR electrical stimulations[Title/Abstract] OR electric stimulations[Title/Abstract] OR electric stimulation[Title/Abstract] OR electro stimulation[Title/Abstract] OR electrostimulus[Title/Abstract] OR galvanostimulation[Title/Abstract] OR electroshocks[Title/Abstract] OR electroshock[Title/Abstract] OR needl*[Title/Abstract] OR chronaxie[Title/Abstract] OR chronaxy[Title/Abstract]

#6 #4 OR #5

#7 "Clinical Trials, Phase II as Topic"[Mesh] OR "Clinical Trials, Phase III as Topic"[Mesh] OR "Clinical Trials, Phase IV as Topic"[Mesh] OR "Controlled Clinical Trials as Topic"[Mesh] OR "Randomized Controlled Trials as Topic"[Mesh] OR "Intention to Treat Analysis"[Mesh] OR "Pragmatic Clinical Trials as Topic"[Mesh] OR "Clinical Trials, Phase II"[Publication Type] OR "Clinical Trials, Phase III"[Publication Type] OR "Clinical Trials, Phase IV"[Publication Type] OR "Controlled Clinical Trials"[Publication Type] OR "Randomized Controlled Trials"[Publication Type] OR "Pragmatic Clinical Trials as Topic"[Publication Type] OR "Single-Blind Method"[Mesh] OR "Double-Blind Method"[Mesh]

#8 random*[Title/Abstract] OR blind*[Title/Abstract] OR singleblind*[Title/Abstract] OR doubleblind*[Title/Abstract] OR trebleblind* [Title/Abstract] OR tripleblind*[Title/Abstract]

#9 #7 OR #8

#10 #3 AND #6 AND #9

Cochrane Library

#1 MeSH descriptor: [Rhinitis, Allergic] explode all trees

#2 MeSH descriptor: [Rhinitis, Allergic, Perennial] explode all trees

#3 MeSH descriptor: [Rhinitis, Allergic, Seasonal] explode all trees

#4 allergic rhinitides:ti,ab,kw or allergic rhinitis:ti,ab,kw or allergic rhinopathy:ti,ab,kw or eosinophil rhinitis:ti,ab,kw or eosinophile rhinitis:ti,ab,kw or eosinophilic rhinitis:ti,ab,kw or eosinophilous rhinitis:ti,ab,kw or allergica rhinitis:ti,ab,kw or eosinophila rhinitis:ti,ab,kw or pollen allergy:ti,ab,kw or pollen allergies:ti,ab,kw or pollinosis:ti,ab,kw or pollinoses:ti,ab,kw or hay fever:ti,ab,kw or hayfever:ti,ab,kw or pollen hypersensitivity:ti,ab,kw or pollen sensitivity:ti,ab,kw or pollenosis:ti,ab,kw or pollinose:ti,ab,kw or seasonal rhinitis:ti,ab,kw

#5 #1 or #2 or #3 or #4

#6 MeSH descriptor: [Acupuncture] explode all trees

#7 MeSH descriptor: [Electric Stimulation] explode all trees

#8 MeSH descriptor: [Chronaxy] explode all trees

#9 MeSH descriptor: [Electroshock] explode all trees

#10 acupuncture:ti,ab,kw or electrical stimulation:ti,ab,kw or electrical stimulations:ti,ab,kw or electric stimulations:ti,ab,kw or electric stimulation:ti,ab,kw or electro stimulation:ti,ab,kw or electrostimulus:ti,ab,kw or galvanostimulation:ti,ab,kw or electroshocks:ti,ab,kw or electroshock:ti,ab,kw or needl*:ti,ab,kw or chronaxie:ti,ab,kw or chronaxy:ti,ab,kw

#11 #6 or #7 or #8 or #9 or #10

#12 #5 and #11

EMBASE

#1 'allergic rhinitis'/exp or 'perennial rhinitis'/exp or 'pollen allergy'/exp

#2 'allergic rhinitides':ti,ab or 'allergic rhinitis':ti,ab or 'allergic rhinopathy':ti,ab or 'eosinophil rhinitis':ti,ab or 'eosinophile rhinitis':ti,ab or 'eosinophilic rhinitis':ti,ab or 'eosinophilous rhinitis':ti,ab or 'allergica rhinitis':ti,ab or 'eosinophila rhinitis':ti,ab or 'pollen allergy':ti,ab or 'pollen allergies':ti,ab or pollinosis:ti,ab or pollinoses:ti,ab or 'hay fever':ti,ab or hayfever:ti,ab or 'pollen hypersensitivity':ti,ab or 'pollen sensitivity':ti,ab or pollenosis:ti,ab or pollinose:ti,ab or 'seasonal rhinitis':ti,ab

#3 #1 or #2

#4 'acupuncture'/exp OR 'electrostimulation'/exp or 'catgut embedding'/exp or 'electroacupuncture'/exp or 'electrostimulation therapy'/exp

#5 acupuncture:ti,ab or 'electrical stimulation':ti,ab or 'electrical stimulations':ti,ab or 'electric stimulations':ti,ab or 'electric timulation':ti,ab or 'electro stimulation':ti,ab or electrostimulus:ti,ab or galvanostimulation:ti,ab or electroshocks:ti,ab or electroshock:ti,ab or needl*:ti,ab or chronaxie:ti,ab or chronaxy:ti,ab

#6 #4 or #5

#7 'multicenter study (topic)'/exp or 'phase 2 clinical trial (topic)'/exp or 'phase 3 clinical trial (topic)'/exp or 'phase 4 clinical trial (topic)'/exp or 'controlled clinical trial (topic)'/exp or 'randomized controlled trial (topic)'/exp or 'single blind procedure'/exp or 'double blind procedure'/exp

#8 random*:ab,ti or blind*:ab,ti or singleblind*:ab,ti or doubleblind*:ab,ti or trebleblind*:ab,ti or tripleblind*:ab,ti

#9 #7 or #8

CBM

("变应性鼻炎"[常用字段] OR "过敏性鼻炎"[常用字段] OR "变态反应性鼻炎"[常用字段]) AND ("针刺"[全字段] OR "针刺"[主题词])
