# Supplementary material for: Acupuncture for allergic rhinitis: a systematic review and meta-analysis
Source: Eur J Med Res. 2022 Apr 25;27:58. doi: 10.1186/s40001-022-00682-3 (PMC9036742; doi:10.1186/s40001-022-00682-3)
Supplement: Supplementary file 3 — Additional file 3. Meta-analysis result for this review. [file 40001_2022_682_MOESM3_ESM.doc]

# Additional file 3

# Result of meta-analysis

## 1. Acupuncture vs waitlist

| **Outcome or Subgroup** | **Studies** | **Participants** | **Statistical Method** | **Effect Estimate** |
| --- | --- | --- | --- | --- |

| 1.1 Nasal symptom: TNSS, higher = severer | 1 | 137 | Mean Difference (IV, Random, 95% CI) | -2.92 [-3.98, -1.86] |
| --- | --- | --- | --- | --- |

| 1.2 Immune response: various serum molecule’s level | 1 | 66 | Mean Difference (IV, Random, 95% CI) | -114.24 [-323.03, 94.55] |
| --- | --- | --- | --- | --- |
| 1.2.1 IgE (kU/L) | 1 | 66 | Mean Difference (IV, Random, 95% CI) | -114.24 [-323.03, 94.55] |

## 2. Acupuncture vs sham acupuncture

| **Outcome or Subgroup** | **Studies** | **Participants** | **Statistical Method** | **Effect Estimate** |
| --- | --- | --- | --- | --- |

| 2.1 Adverse events | 3 |  | Risk Ratio (M-H, Random, 95% CI) | Subtotals only |
| --- | --- | --- | --- | --- |
| 2.1.1 Any adverse event | 2 | 363 | Risk Ratio (M-H, Random, 95% CI) | 1.16 [0.63, 2.13] |
| 2.1.2 mild discomfort | 1 | 80 | Risk Ratio (M-H, Random, 95% CI) | 1.24 [0.56, 2.76] |
| 2.1.3 mild headache | 1 | 80 | Risk Ratio (M-H, Random, 95% CI) | 0.45 [0.04, 4.79] |
| 2.1.4 mild dizziness | 1 | 80 | Risk Ratio (M-H, Random, 95% CI) | 2.72 [0.11, 64.85] |
| 2.1.5 pain at needling area after treatment | 1 | 175 | Risk Ratio (M-H, Random, 95% CI) | 3.21 [1.09, 9.47] |
| 2.1.6 Bruise | 1 | 175 | Risk Ratio (M-H, Random, 95% CI) | 6.92 [0.87, 55.08] |
| 2.1.7 numbness and weakness at local area | 1 | 175 | Risk Ratio (M-H, Random, 95% CI) | 4.94 [0.24, 101.51] |
| 2.1.8 feeling tired after needling | 1 | 175 | Risk Ratio (M-H, Random, 95% CI) | 2.97 [0.12, 71.83] |
| 2.1.9 feeling nausea after needling | 1 | 175 | Risk Ratio (M-H, Random, 95% CI) | 2.97 [0.12, 71.83] |
| 2.1.10 Headache | 1 | 175 | Risk Ratio (M-H, Random, 95% CI) | 0.33 [0.07, 1.59] |
| 2.1.11 Low mood | 1 | 175 | Risk Ratio (M-H, Random, 95% CI) | 0.33 [0.01, 7.98] |
| 2.1.12 constipation | 1 | 175 | Risk Ratio (M-H, Random, 95% CI) | 0.33 [0.01, 7.98] |

## 3. Acupuncture vs Cetirizine

| **Outcome or Subgroup** | **Studies** | **Participants** | **Statistical Method** | **Effect Estimate** |
| --- | --- | --- | --- | --- |

| 3.1 Nasal symptoms: TNSS, higher = severe | 3 | 214 | Mean Difference (IV, Random, 95% CI) | -0.77 [-1.67, 0.12] |
| --- | --- | --- | --- | --- |

| 3.2 Immune response: various serum moleculers level | 3 | 610 | Mean Difference (IV, Random, 95% CI) | -6.62 [-13.04, -0.21] |
| --- | --- | --- | --- | --- |
| 3.2.1 IgE (U/mL) | 2 | 331 | Mean Difference (IV, Random, 95% CI) | -52.83 [-78.78, -26.88] |
| 3.2.3 IL-4 (ng/mL) | 1 | 93 | Mean Difference (IV, Random, 95% CI) | 0.02 [-0.21, 0.25] |
| 3.2.4 INF gama (pg/mL) | 1 | 93 | Mean Difference (IV, Random, 95% CI) | 1.72 [-0.17, 3.60] |

## 4. Acupuncture vs Loratadine

| **Outcome or Subgroup** | **Studies** | **Participants** | **Statistical Method** | **Effect Estimate** |
| --- | --- | --- | --- | --- |

| 4.1 Nasal symptoms: TNSS, higher = severe | 1 | 75 | Mean Difference (IV, Random, 95% CI) | -1.75 [-2.66, -0.84] |
| --- | --- | --- | --- | --- |

| 4.2 Relapse at 1 year | 1 | 26 | Risk Ratio (M-H, Random, 95% CI) | 0.38 [0.16, 0.93] |
| --- | --- | --- | --- | --- |

## 5. Acupuncture vs Terfenadine

| **Outcome or Subgroup** | **Studies** | **Participants** | **Statistical Method** | **Effect Estimate** |
| --- | --- | --- | --- | --- |

| 5.1 Clinical response: various criteria | 1 | 124 | Risk Ratio (M-H, Random, 95% CI) | 1.14 [1.00, 1.30] |
| --- | --- | --- | --- | --- |
| 5.1.3 other subjective criteria | 1 | 124 | Risk Ratio (M-H, Random, 95% CI) | 1.14 [1.00, 1.30] |

## 6. Acupuncture vs Tranilast Capsules

| **Outcome or Subgroup** | **Studies** | **Participants** | **Statistical Method** | **Effect Estimate** |
| --- | --- | --- | --- | --- |

| 6.1 Clinical response: various criteria | 1 | 386 | Risk Ratio (M-H, Random, 95% CI) | 1.34 [1.21, 1.48] |
| --- | --- | --- | --- | --- |
| 6.1.2 TNSS>=20% | 1 | 386 | Risk Ratio (M-H, Random, 95% CI) | 1.34 [1.21, 1.48] |

## 7. Acupuncture vs Desloratadine dispersible

| **Outcome or Subgroup** | **Studies** | **Participants** | **Statistical Method** | **Effect Estimate** |
| --- | --- | --- | --- | --- |

| 7.1 Clinical response: various criteria | 1 | 66 | Risk Ratio (M-H, Random, 95% CI) | 1.29 [1.03, 1.62] |
| --- | --- | --- | --- | --- |
| 7.1.2 TNSS>=20% | 1 | 66 | Risk Ratio (M-H, Random, 95% CI) | 1.29 [1.03, 1.62] |

| 7.2 Nasal symptoms: TNSS, higher = severe | 1 | 66 | Mean Difference (IV, Random, 95% CI) | -1.85 [-3.01, -0.69] |
| --- | --- | --- | --- | --- |

## 8. Acupuncture + western medicine vs western medicine alone

| **Outcome or Subgroup** | **Studies** | **Participants** | **Statistical Method** | **Effect Estimate** |
| --- | --- | --- | --- | --- |

| 8.1 Clinical response: various criteria | 3 | 277 | Risk Ratio (M-H, Random, 95% CI) | 1.21 [1.02, 1.43] |
| --- | --- | --- | --- | --- |
| 8.1.1 decrease rate TNSS>=25% | 2 | 176 | Risk Ratio (M-H, Random, 95% CI) | 1.13 [1.02, 1.24] |
| 8.1.2 TNSS>=20% | 1 | 101 | Risk Ratio (M-H, Random, 95% CI) | 1.49 [1.19, 1.86] |

| 8.2 Nasal symptoms: RQLQ nasal symptoms, higher = severer | 1 | 320 | Mean Difference (IV, Random, 95% CI) | -0.70 [-1.12, -0.28] |
| --- | --- | --- | --- | --- |

| 8.3 Adverse events | 1 |  | Risk Ratio (M-H, Random, 95% CI) | Subtotals only |
| --- | --- | --- | --- | --- |
| 8.3.1 Any adverse event | 1 | 76 | Risk Ratio (M-H, Random, 95% CI) | 0.54 [0.22, 1.34] |
| 8.3.2 fatigue | 1 | 76 | Risk Ratio (M-H, Random, 95% CI) | 0.05 [0.00, 0.89] |
| 8.3.3 limb joint pain | 1 | 76 | Risk Ratio (M-H, Random, 95% CI) | 0.30 [0.01, 7.16] |

## 9. Acupuncture vs sham acupuncture in Children

| **Outcome or Subgroup** | **Studies** | **Participants** | **Statistical Method** | **Effect Estimate** |
| --- | --- | --- | --- | --- |

| 9.1 Nasal symptoms: Daily rhinitis scores, higher = severer | 1 | 72 | Mean Difference (IV, Random, 95% CI) | -1.76 [-3.59, 0.07] |
| --- | --- | --- | --- | --- |

| 9.2 Adverse events | 1 | 72 | Risk Ratio (M-H, Random, 95% CI) | 1.32 [0.39, 4.52] |
| --- | --- | --- | --- | --- |

Note: TNSS = Total Nasal Symptom Score; RQLQ = Rhinoconjunctivitis Quality of Life Questionnaire; IV = Inverse Variance; CI = Confidence Interval.
