# Supplementary material for: Acupuncture for allergic rhinitis: a systematic review and meta-analysis
Source: Eur J Med Res. 2022 Apr 25;27:58. doi: 10.1186/s40001-022-00682-3 (PMC9036742; doi:10.1186/s40001-022-00682-3)
Supplement: Supplementary file 4 — Additional file 4. Result of subgroup analysis according to different time-points and countries. [file 40001_2022_682_MOESM4_ESM.doc]

## SUBGROUP ANALYSIS 1: Acupuncture vs waitlist

| **Outcome or Subgroup** | **Studies** | **Participants** | **Statistical Method** | **Effect Estimate** |
| --- | --- | --- | --- | --- |

| 1.1 Quality of life: RQLQ, lower = better, different time points | 3 | 1112 | Standard Mean Difference (IV, Random, 95% CI) | -0.95 [-1.17, -0.73] |
| --- | --- | --- | --- | --- |
| 1.1.1 4 weeks | 1 | 137 | Mean Difference (IV, Random, 95% CI) | -0.95 [-1.24, -0.66] |
| 1.1.2 8 weeks | 1 | 73 | Mean Difference (IV, Random, 95% CI) | -10.06 [-17.89, -2.23] |
| 1.1.3 3 months | 1 | 902 | Mean Difference (IV, Random, 95% CI) | -0.90 [-1.01, -0.79] |

| 1.2 Quality of life: RQLQ, lower = better, different countries | 3 | 1112 | Standard Mean Difference (IV, Random, 95% CI) | -0.95 [-1.17, -0.73] |
| --- | --- | --- | --- | --- |
| 1.2.1 German | 1 | 902 | Mean Difference (IV, Random, 95% CI) | -0.90 [-1.01, -0.79] |
| 1.2.2 China and Korea | 1 | 137 | Mean Difference (IV, Random, 95% CI) | -0.95 [-1.24, -0.66] |
| 1.2.3 Australia | 1 | 73 | Mean Difference (IV, Random, 95% CI) | -10.06 [-17.89, -2.23] |

## SUBGROUP ANALYSIS 2: Acupuncture vs sham acupuncture

| **Outcome or Subgroup** | **Studies** | **Participants** | **Statistical Method** | **Effect Estimate** |
| --- | --- | --- | --- | --- |

| 2.1 Nasal symptoms: RQLQ nasal symptoms, higher = severer, different time points | 2 | 489 | Mean Difference (IV, Random, 95% CI) | -0.60 [-1.16, -0.04] |
| --- | --- | --- | --- | --- |
| 2.1.1 4 weeks | 1 | 175 | Mean Difference (IV, Random, 95% CI) | -1.41 [-3.04, 0.22] |
| 2.1.2 8 weeks | 1 | 314 | Mean Difference (IV, Random, 95% CI) | -0.50 [-0.92, -0.08] |

| 2.2 Nasal symptoms: RQLQ nasal symptoms, higher = severer, different countries | 2 | 489 | Mean Difference (IV, Random, 95% CI) | -0.60 [-1.16, -0.04] |
| --- | --- | --- | --- | --- |
| 2.2.1 Australia | 1 | 175 | Mean Difference (IV, Random, 95% CI) | -1.41 [-3.04, 0.22] |
| 2.2.2 German | 1 | 314 | Mean Difference (IV, Random, 95% CI) | -0.50 [-0.92, -0.08] |

| 2.3 Quality of life: RQLQ, lower = better, different time points | 3 | 436 | Std. Mean Difference (IV, Random, 95% CI) | -0.26 [-0.44, -0.07] |
| --- | --- | --- | --- | --- |
| 2.3.1 4 weeks | 2 | 363 | Std. Mean Difference (IV, Random, 95% CI) | -0.22 [-0.43, -0.01] |
| 2.3.2 8 weeks | 1 | 73 | Std. Mean Difference (IV, Random, 95% CI) | -0.43 [-0.89, 0.03] |

| 2.4 Quality of life: RQLQ, lower = better, different countries | 3 | 436 | Std. Mean Difference (IV, Random, 95% CI) | -0.26 [-0.44, -0.07] |
| --- | --- | --- | --- | --- |
| 2.4.1 Korea and China | 1 | 188 | Std. Mean Difference (IV, Random, 95% CI) | -0.17 [-0.46, 0.11] |
| 2.4.2 Australia | 2 | 248 | Std. Mean Difference (IV, Random, 95% CI) | -0.32 [-0.57, -0.07] |

## SUBGROUP ANALYSIS 3: Acupuncture vs Cetirizine

| **Outcome or Subgroup** | **Studies** | **Participants** | **Statistical Method** | **Effect Estimate** |
| --- | --- | --- | --- | --- |
| 3.1 Clinical response: various criteria | 5 | 588 | Risk Ratio (M-H, Random, 95% CI) | 1.10 [0.96, 1.26] |
| 3.1.1 4 weeks | 4 | 522 | Risk Ratio (M-H, Random, 95% CI) | 1.13 [0.95, 1.35] |
| 3.1.2 8 weeks | 1 | 66 | Risk Ratio (M-H, Random, 95% CI) | 1.01 [0.86, 1.17] |

| 3.2 Nasal symptoms: TNSS, higher = severe, different time points | 3 | 214 | Mean Difference (IV, Random, 95% CI) | -0.77 [-1.67, 0.12] |
| --- | --- | --- | --- | --- |
| 3.2.1 4 weeks | 2 | 148 | Mean Difference (IV, Random, 95% CI) | -0.47 [-2.68, 1.74] |
| 3.2.2 8 weeks | 1 | 66 | Mean Difference (IV, Random, 95% CI) | -0.78 [-1.37, -0.19] |

## SUBGROUP ANALYSIS 4: Acupuncture vs Loratadine

| **Outcome or Subgroup** | **Studies** | **Participants** | **Statistical Method** | **Effect Estimate** |
| --- | --- | --- | --- | --- |

| 4.1 Clinical response: other subjective criteria, different time points | 6 | 333 | Risk Ratio (M-H, Random, 95% CI) | 1.15 [0.98, 1.37] |
| --- | --- | --- | --- | --- |
| 4.1.1 2 weeks | 1 | 60 | Risk Ratio (M-H, Random, 95% CI) | 0.96 [0.83, 1.12] |
| 4.1.2 4 weeks | 4 | 249 | Risk Ratio (M-H, Random, 95% CI) | 1.21 [0.99, 1.48] |
| 4.1.3 6 weeks | 1 | 24 | Risk Ratio (M-H, Random, 95% CI) | 1.30 [0.79, 2.15] |
| 4.2 Clinical response: other subjective criteria, different countries | 6 | 333 | Risk Ratio (M-H, Random, 95% CI) | 1.15 [0.98, 1.37] |
| 4.2.1 China | 5 | 309 | Risk Ratio (M-H, Random, 95% CI) | 1.15 [0.95, 1.37] |
| 4.2.3 German | 1 | 24 | Risk Ratio (M-H, Random, 95% CI) | 1.30 [0.79, 2.15] |
